# Association of internalised homonegativity with partner notification after diagnosis of syphilis or gonorrhoea among men having sex with men in 49 countries across four continents

**DOI:** 10.1186/s12889-022-14891-2

**Published:** 2023-01-03

**Authors:** Ulrich Marcus, Kai Jonas, Rigmor Berg, Maria Amelia Veras, Carlos F. Caceres, Jordi Casabona, Susanne B. Schink, Axel J. Schmidt

**Affiliations:** 1 Department of Infectious Disease Epidemiology, Robert Koch Institute, Berlin, Germany; 2 Faculty of Psychology and Neuroscience, Maastricht University, Maastricht, The Netherlands; 3 Division for the Health Services, Norwegian Institute of Public Health, Oslo, Norway; 4 Department of Community Medicine, University of Tromso, Tromso, Norway; 5 Faculdade de Ciências Médicas da Santa Casa de São Paulo, Rua Dr Cesario Mota Jr 61, São Paulo, SP 01221-020 Brazil; 6 Centre for Interdisciplinary Studies in Sexuality, AIDS and Society, Universidad Peruana Cayetano Heredia, Lima, Peru; 7 Health Department, Centre of Epidemiological Studies of HIV/AIDS and STI of Catalonia (CEEISCAT), Generalitat de Catalunya, Badalona, Spain; 8 Germans Trias I Pujol Research Institute (IGTP), Campus Can Ruti, Badalona, Spain; 9 CIBER Epidemiologia Y Salud Pública (CIBERESP), Madrid, Spain; 10 Department of Paediatrics, Obstetrics and Gynecology and Preventive Medicine, Universitat Autònoma de Barcelona, Badalona, Spain; 11 Department of Public Health, Environments and Society, London School of Hygiene and Tropical Medicine, Sigma Research, London, UK

**Keywords:** Contact tracing, Syphilis, Gonorrhoea, Sexual and gender minorities, Internalised homonegativity, Regression analysis, Europe, Latin America, Canada, Philippines

## Abstract

**Background:**

Partner notification (PN) after a sexually transmitted infection (STI) diagnosis is being promoted as a means to interrupt transmission chains. We investigated whether Internalised Homonegativity (IH) is associated with PN among men having sex with men (MSM).

**Methods:**

PN, defined as notifying at least one partner after diagnosis of syphilis and gonorrhoea, was queried in two internet-based self-completion surveys conducted between Oct 2017 and May 2018 in 68 countries in Europe, Latin America, Canada, and the Philippines. IH is defined by a man’s level of agreement or disagreement with negative social beliefs about male homosexuality. Covariates included in a multivariate regression model with a random intercept at country level were age, HIV diagnosis, partnership status, sexual self-efficacy, HIV serostatus communication during last sex with a non-steady partner, place where this partner was met, and PN-related socio-historical background of the country of residence. We grouped countries in three areas: North- and Central-Western European countries plus Canada, former socialist countries, and Latin-American/Mediterranean countries plus the Philippines. In each of the three areas individuals were assigned to 4 subgroups based on IH quartiles and PN rates were determined for each subgroup.

**Results:**

PN rates were calculated for 49 countries (excluding countries with less than 10 diagnoses). Mean proportions of MSM notifying their partners were 68.1% and 72.9% after syphilis and gonorrhoea diagnoses, respectively. PN rates were lower in Latin American countries and the Philippines compared to European countries. Within Europe, a North–South divide with lower PN rates in Mediterranean countries was observed. In each of the three regions we mostly observed a stepwise increase of PN rates with decreasing IH.

Regression analysis showed lower IH scores associated with higher PN rates. Higher perceived self-efficacy, living in a partnership, and HIV status communication were positively associated with PN. Men who had met their last partner in a gay social venue were more likely to have notified their partners of a syphilis diagnosis compared to men who had met this partner online. Men with diagnosed HIV were less likely to report PN.

**Conclusions:**

We could demonstrate that IH was associated with PN among MSM across all countries included in our analysis. Reducing cultural homophobia and ensuring inclusive policies may contribute to STI prevention and control.

**Supplementary Information:**

The online version contains supplementary material available at 10.1186/s12889-022-14891-2.

## Background

Partner notification (PN) after the diagnosis of an acute sexually transmitted infection (STI) has long been advocated for as a means to interrupt transmission chains. PN alerts sex partners of their exposure and allows them to get treated and/or tested for STIs without delay, and furthermore prevents reinfection from the same partners. Partners can be informed by the patient (patient referral) or by a health professional (provider referral). In practice, patient referral is the most commonly used method [1, 2]. Implementation of PN varies considerably across countries. In most countries, it is recommended while some countries mandate PN by law (usually without any means of enforcement) and some provide dedicated notification services [1, 2].

Individuals diagnosed with an STI can be in different partnerships simultaneously, with different patterns of communication, perceived exposure risk, and anticipated consequences of notification or concealment. For all individuals, PN involves disclosure of private and oftentimes secret information that could impact a partnership [3]. Sexual orientation stigmatization poses another hurdle for men who have sex with men (MSM) and other sexual minorities. In addition to informing partners of an STI diagnosis, which can be difficult in itself, PN may reveal other potentially stigmatizing information related to sexual orientation and sexual practices, and frequently changing partners.

Previous work showed that notification and communication about potential STI exposures are determined by the type of partnership, the length of the relationship, standards of communication between partners, and/or availability of contact information [4–8]. In steady partnerships, open communication is often assumed. In contrast, an absence of emotional commitment with non-steady or anonymous partners is often coupled with a lack of communication and a reduced sense of responsibility for these partners’ well-being. For anonymous encounters, the lack of contact information precludes notification. However, partners first met online can often also be notified online while keeping their identity anonymous.

Previous studies have addressed individual-, partner-, and network-level pathways that define notification outcomes. In terms of individual behaviour, issues of self-efficacy and empowerment have been shown to influence notification behaviours [9–12]. Schwartz et al. found that a positive intention to notify partners was a significant predictor of notification. They confirmed associations between self-efficacy, attitudes, and behavioural intentions; higher levels of self-efficacy and more positive attitudes about notification were associated with greater intention to notify partners [13]. Being out about ones’ sexual orientation and low internalised homonegativity (IH) have been shown to be beneficial for successful and open communication between homosexual partners [14–16]. IH refers to negative attitudes that MSM may have towards homosexuality in general, and towards their own sexual orientation [17] and the degree to which those beliefs affect the development of an affirmative sexual identity [18]. Homophobic policies and a homophobic socio-cultural environment are strongly related to structural stigma and increased IH [19, 20]. Low IH has been shown to be likely related to increased self-esteem and a sense of connectedness within sexual minority communities; both are factors that have previously been shown to be associated with improved health outcomes [21, 22]. Several studies suggested that IH interferes with sexual minority identity formation, impacts on openness about sexual orientation, disclosure, and gay community connection and gay social support overall [23, 24]. However, few studies to date have explored the effect of psychosocial constructs such as IH on PN activities of MSM, and none have focused on the association of IH with PN among MSM [25].

Historically, PN or ‘contact tracing’ was rigorous in the Soviet Union and most former socialist countries in central and eastern Europe [26, 27]. The Scandinavian countries, the United Kingdom (UK), and Canada are known to encourage PN and have established PN support systems. A survey conducted in 1998 – 99 reported that PN at that time was not part of STI case management in France, Spain, and Italy [28], indicating large cultural differences regarding the practice of STI PN in Europe. Garcia et al. reported in an overview on STI management and control in Latin America that PN rates in Latin America are usually low [29]. Given historically determined differences across countries, it is important to update geo-cultural determinants as well.

We set out to analyse the role of IH for self-reported PN after a diagnosis of syphilis or gonorrhoea in two large datasets of MSM living in Europe, Latin America, Canada, and the Philippines. We would expect that IH might influence the extent of PN among MSM via its impact on perceived self-efficacy, ability to communicate with sexual partners, and a higher sense of connectedness.

## Methods

### Recruitment and surveys

The detailed methods of the European MSM Internet Survey 2017 (EMIS) and the corresponding Latin American survey 2018 (LAMIS) have been reported elsewhere [30, 31]. In summary, both were multi-language, internet-based, self-completion surveys for MSM living in Europe and in Latin America. Recruitment was from 13 Oct 2017 to 31 Jan 2018 (EMIS) and from 24 Jan to 13 May 2018 (LAMIS). The EMIS data collection additionally included a few non-European countries, namely Israel, Lebanon, Canada, and the Philippines. EMIS and LAMIS were nearly identical in use of instruments and questions to collect data about morbidities, behaviours, needs and interventions. Regarding morbidities, respondents were asked about diagnoses of HIV, syphilis, gonorrhoea, and chlamydia. In terms of behaviours, the respondents were asked about communication about HIV status and use of antiretroviral drugs with their last non-steady partner(s), where they had met this/these partner(s), and how many of the people they knew were aware of their sexual attraction to men because they are open about sexual identity (outness). There were two questions about sexual self-efficacy. We measured IH with the Short Internalised Homonegativity Scale (SIHS), based on seven statements [32]. While the scale was presented to half of the EMIS respondents (at random, to keep the questionnaire short), all LAMIS respondents were presented the scale.

EMIS was available in 33 languages across 50 countries, LAMIS in three languages across 18 countries. Participants were recruited through trans-national dating apps (PlanetRomeo, Grindr and Hornet accounted for 69% of participants to both surveys collectively, other dating platforms and apps for another 9%), through Facebook, Twitter, Instagram (8%), and through a variety of local online promotion, mostly through website banners (8%). No financial incentives were given to participants. No personal identifying information (including IP addresses) was collected. Further background information, including all 33 language versions of the questionnaires, is available at www.emis2017.eu. Ethics approval was granted by the Ethics Committee of the London School of Hygiene and Tropical Medicine for EMIS-2017 (reference 14421/RR/8805), and by the committees of the Universidad Peruana Cayetano Heredia (612–19-17), the Salvador Allende School of Public Health, Faculty of Medicine, University of Chile (009–2017), Santa Casa de Misericórdia de São Paulo, Brazil (2,457,744), the National Committee for Health Ethics, Guatemala (39–2017), and the Faculty of Psychology and Neuroscience of the University of Maastricht, the Netherlands (18–01-12–2017) for LAMIS.

### Dependent variables

We had two primary outcomes: partner notification for (1) syphilis and (2) gonorrhoea. All respondents were asked “*Have you ever been diagnosed with syphilis?*” Men who answered yes, were asked “*When were you last diagnosed with syphilis?*” and offered answers to indicate how recently this had been. Matching questions were asked for gonorrhoea. Those who reported a diagnosis of syphilis or gonorrhoea were asked: “*The last time you were diagnosed with [syphilis/gonorrhoea], did you (or your healthcare provider) inform your recent sexual partners that they also needed a test/treatment?*” Response options were “No, none of them”, “Yes, some of them”, “Yes, all of them”, and “I don’t remember”. The two “Yes” options and the first and last responses were combined into a binary outcome variable to measure the association between IH and notification of at least one partner.

### Independent variables

#### Internalised Homonegativity

IH was measured by a continuous scale composed of seven statements. Each of them could be rated on a seven-point agreement scale (0–6) and an additional “does not apply to me” response option [32] (see also Supplemental Table S1).

#### Sexual self-efficacy

Self-efficacy was measured by a 5-point disagree-agree response to the statement “The sex I have is always as safe as I want it to be”. Responses were dichotomized into a binary variable for this analysis, combining neutral and two “agree”-options vs. the two “disagree” options**.**

#### Behaviours

Hypothesizing that previous communication with non-steady sex partners about sensitive issues would affect PN after an STI diagnosis, we constructed a binary variable: any HIV status communication vs. none, based on HIV serostatus communications during the last sexual encounter with one or more non-steady partners in the previous twelve months. Respondents not reporting non-steady partner(s) in the previous twelve months were classified as “no serostatus communication”. Depending on the place where the last non-steady sex partners were met, we constructed a variable with the categories “online”, “gay sex venue”, “social venue”, and “no non-steady partner sex or not answered”.

#### Survey artefacts

The wording for the French translation for STI diagnoses, while technically correct, may have been misunderstood by some European French-speaking respondents: the questions on diagnosed syphilis and gonorrhoea may have been understood by some men as having been tested rather than having received a positive test result. This problem affected all three countries with large sub-samples using the French questionnaire, notably France (93%), Belgium (36%), and Switzerland (19%). All respondents who answered affirmatively to the question were asked about PN. Since respondents who had only been tested and not been diagnosed with syphilis or gonorrhoea would feel no need to inform their partners, a larger proportion would be expected to report no PN. To control for a potential underestimation of PN in surveys completed in French, a binary language variable (French – not French) was constructed. We further controlled for major discrepancies (discrepant answers for age, steady partners, or non-steady partners), using a binary variable. Such discrepancies occur when respondents either give random answers or always select the first response option.

Countries with less than 10 respondents diagnosed with syphilis or gonorrhoea were excluded from our analysis.

#### Sample composition

In the multivariable regression models age was included as age groups < 25, 25–39, 40 + . HIV diagnosis was included as a binary variable. Partnership status was categorized as “single”, “having a steady partner”, and “not sure / it’s complicated”.

#### Grouping of individuals by cultural/socio-historical background of the country of residence and IH quartile

To determine whether the impact of IH on PN was independent of the broader cultural/socio-historical background we grouped individuals by three areas with similar socio-historical backgrounds regarding STI PN, i.e. North- and Central-Western European countries plus Canada, former socialist countries, Latin American/Mediterranean countries plus the Philippines, which we found to empirically cluster in terms of PN rates, with each 4 subgroups (based on IH quartiles). Individuals’ characteristics on these two variables resulted in 12 groups.

### Statistical analysis

For continuous variables mean, Standard Deviation (SD), median and interquartile range (IQR) were used. For nominal variables count and percentages were used.

Based on literature, a list of variables, potentially associated with the dependent variables, was developed for both outcome variables. Besides IH, which was the primary focus of our analysis, the covariates included a question on sexual self-efficacy, HIV diagnosis, current partnership status, HIV serostatus communication with the last non-steady partner, and meeting place of the last non-steady partner. The last two variables not necessarily refer to the partner(s) who have been notified of the reported STI diagnosis. However, supported by published findings, we assume that individual behaviours with the last non-steady partner represent a more general behaviour pattern at a population level [33, 34]. The variables were first tested in a bivariate analysis and then included in a two-level logistic regression model with a random intercept at country level. The random component accounts for the hierarchical nature of the data. All available cases were included in the analysis.

We developed a model for both dependent variables. We then sequentially entered statistically significant (based on bivariate analysis) variables for each model. Age was included as confounder to be controlled for as potentially associated with the outcome variables. The final models were then estimated with the pool of significantly associated variables. We used the likelihood ratio (LR) test to compare the new model with the nested model to establish the model improvement. For all statistical tests, significance was indicated by p < 0.05. The final model estimated the adjusted odds ratios (aORs) and the corresponding 95% confidence interval (95% CI) for factors associated with the dependent variable.

Analyses were carried out using Stata® Version 17.1 (College Station, TX: StataCorp LP), graphs were compiled in R and R Studio 4.1.2 (The R Foundation for Statistical Computing). Maps were generated in EMMa: ECDC Map Maker, European Centre for Disease Prevention and Control, 2022. Administrative boundaries: © EuroGeographics © UN-FAO © Turkstat. The boundaries and names shown on this map do not imply official endorsement or acceptance by the European Union. Map produced on 18 Jul 2022.

## Results

Partner notification rates for syphilis and gonorrhoea could be calculated for 49 countries (excluding 19 countries with less than 10 respondents diagnosed with syphilis or gonorrhoea; see Supplemental Table S2).

For these 49 countries, the country median of MSM who notified their partner(s) about STI diagnosis was 69% for syphilis (range 28.6%–91.0%), and 71.4% for gonorrhoea (range 25.0%–100%). The overall mean proportion of MSM notifying their partners after a syphilis diagnosis across the 49 countries was 68.1%, for gonorrhoea notification the mean was 72.9%. Due to the potentially downward biased PN rates of the three countries with larger proportions of French questionnaires, PN rates for these countries were calculated only based on reported partner notifications in non-French questionnaires.

**Fig.1:**
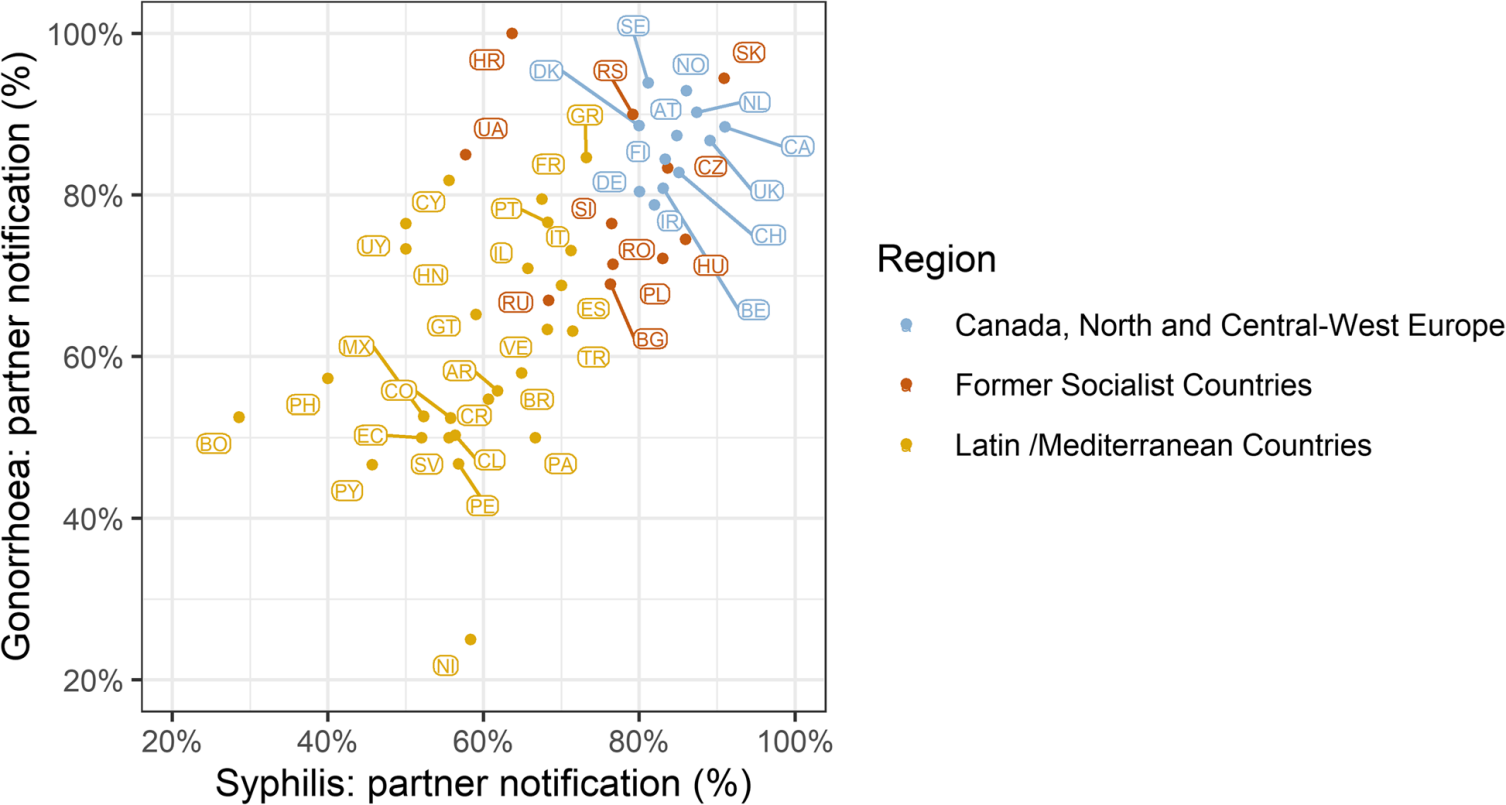
Correlation between syphilis and gonorrhoea partner notification – Scatterplot of 49 countries

The correlation between syphilis and gonorrhoea PN rates was high, but larger discrepancies with lower syphilis PN rates were observed in seven countries (Croatia, Ukraine, Cyprus, Honduras, Uruguay, Philippines, and Bolivia, see Fig. 1). In five of these countries this may be explained by measurement errors due to low overall numbers of diagnoses.

There was a discernible general trend towards lower PN rates in Latin American countries and the Philippines compared to European countries. Within Europe, we observed a North–South divide with lower PN rates in Mediterranean countries such as Turkey, Israel, Greece, Italy, France, Spain, and Portugal.

**Fig.2:**
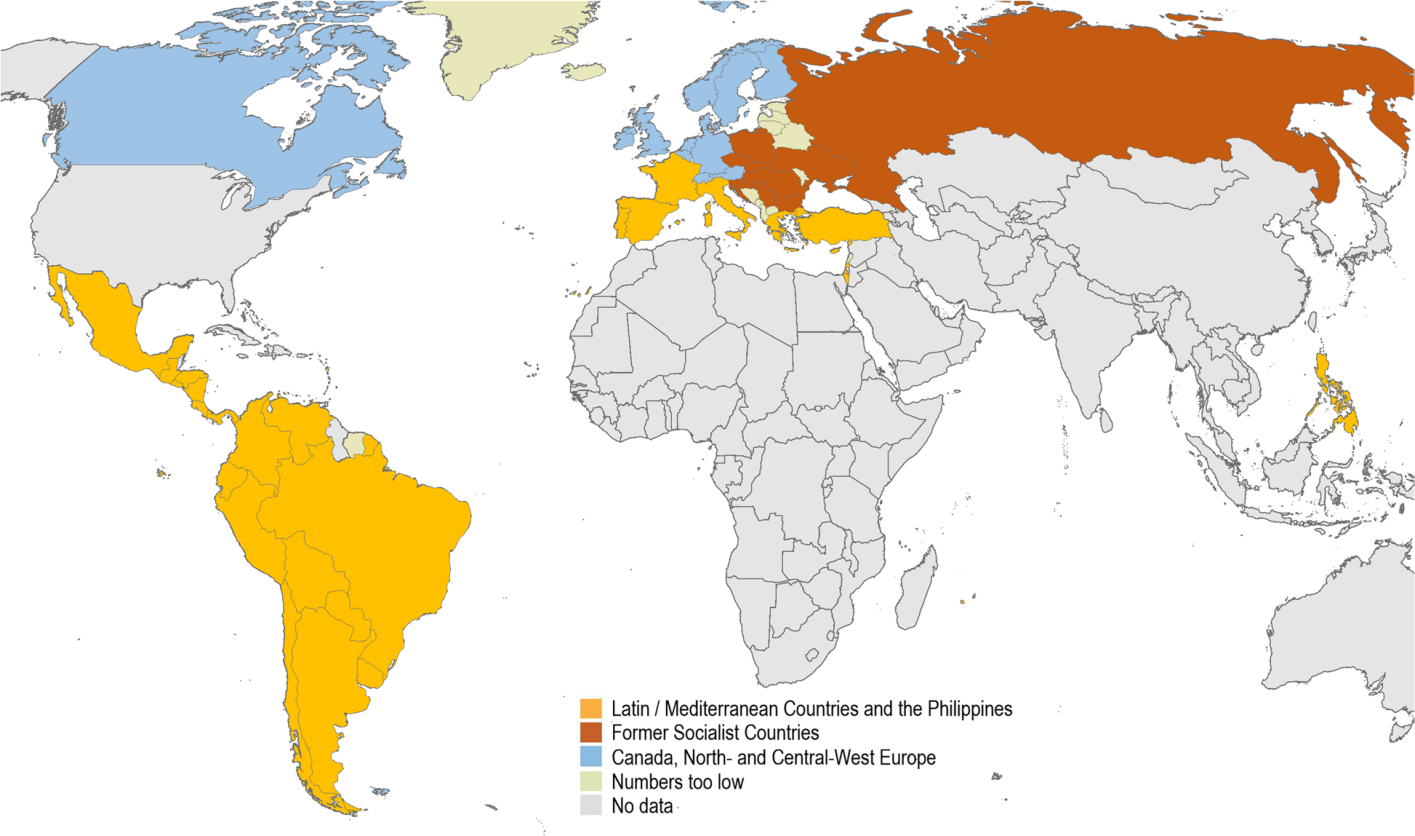
Regional patterns of syphilis and gonorrhoea partner notification by men having sex with men participating in EMIS and LAMIS

The grouping of countries based on cultural patterns of PN and socio-cultural, historical and cultural similarities is shown in Fig. 2: area 1 included countries in Latin America, Western Mediterranean countries where Romanic languages are spoken (Italy, France, Spain, Portugal), Eastern Mediterranean countries (Greece, Turkey, Israel), and the Philippines; area 2 included the former socialist countries in Central-East, East, and South-East Europe; and area 3 included Canada and the remaining countries in North and Central-West Europe. When we grouped individuals by the socio-historical background of the country they lived in and IH quartiles into 12 groups (quartile 1 score 0–0.571, quartile 2 score 0.572–1.286, quartile 3 score 1.287–2.286, and quartile 4 score 2.287–5.143), we saw distinct differences primarily between area 1 and area 2 and 3. In each country area we observed a stepwise increase of PN rates with decreasing IH—except for two strata, one in the former socialist country area, and one in the North and Central-West European area, which included relatively small numbers of individuals (see Table 1).

**Table 1:** Syphilis and gonorrhoea partner notification (PN) by socio-cultural background and internalised homonegativity score quartiles in 49 countries

	**Syphilis-PN**				**Gonorrhoea-PN**			
IH quartiles	No partner notification	Partner notification	Total	**% with PN**	No partner notification	Partner notification	Total	**% with PN**
**Latin/Mediterranean countries**								
0.0–0.57	566	1,023	1,589	**64.4%**	363	646	1.009	**64.0%**
0.57–1.28	422	734	1,156	**63.5%**	249	397	646	**61.5%**
1.28–2.28	569	717	1,286	**55.8%**	319	455	774	**58.8%**
2.28–5.14	672	691	1,363	**50.7%**	463	473	936	**50.5%**
Total	2,229	3,165	5,394	**58.7%**	1,394	1,971	3,365	**58.6%**
**Former socialist countries**								
0.0–0.57	12	39	51	**76.5%**	3	36	39	**92.3%**
0.57–1.28	16	42	58	**72.4%**	7	27	34	**79.4%**
1.28–2.28	23	81	104	**77.9%**	19	58	77	**75.3%**
2.28–5.14	30	58	88	**65.9%**	22	50	72	**69.4%**
Total	81	220	301	**73.1%**	51	171	222	**77.0%**
**Canada, North- and Central-West European countries**								
0.0-–0.57	67	392	459	**85.4%**	117	713	830	**85.9%**
0.57–1.28	44	148	192	**77.1%**	69	337	406	**83.0%**
1.28–2.28	48	154	202	**76.2%**	79	303	382	**79.3%**
2.28–5.14	33	124	157	**79.0%**	57	200	257	**77.8%**
Total	192	818	1,010	**81.0%**	322	1,553	1,875	**82.8%**

*IH* short internalised homonegativity score (range 0–6)

### Multilevel multivariate analysis of factors associated with syphilis PN

Lower IH scores were associated with higher PN rates, as was higher perceived self-efficacy. Living in a steady partnership was positively associated with PN, as was HIV status communication with the last non-steady partner(s). Men with diagnosed HIV were less likely to notify their partners about a syphilis diagnosis. Men who had met their last non-steady partner in a gay social venue were more likely to have notified their partners after having been diagnosed with syphilis compared to men who had met their last non-steady partner(s) online.

Downgrading of PN rates by the French translation issue was confirmed for syphilis. Age was not significant.

The cultural background of the country had a major impact on the reported likelihood to notify partners (see Table 2).

**Table 2:** Uni- and multivariable multilevel regression analysis of variables associated with syphilis and gonorrhoea partner notification (PN) among MSM in 49 countries

	**Syphilis PN (*N* = 6,705)**			**Syphilis PN (*N* = 6,492)**			**Gonorrhoea PN (*N* = 5,462)**			**Gonorrhoea PN (*N* = 5,340)**		
	**OR**	**95% CI**	***p***	**aOR**	**95% CI**	***p***	**OR**	**95% CI**	***p***	**aOR**	**95% CI**	***p***
**French language questionnaire**												
No	ref			ref			ref			ref		
Yes	**0.53**	0.34 – 0.83	0.006	**0.55**	0.36 – 0.82	0.004	0.72	0.47 – 1.11	0.133	0.80	0.53 – 1.21	0.294
**Age**												
< 25	ref			ref						ref		
25–39	1.03	0.90 – 1.18	0.651	1.09	0.95 – 1.26	0.224	1.07	0.93 – 1.25	0.345	1.04	0.89 – 1.22	0.599
40 +	0.97	0.82 – 1.15	0.716	1.01	0.84 – 1.21	0.943	0.85	0.70 – 1.03	0.105	**0.81**	0.66 – 1.00	0.047
**Partnership status**												
No steady partner	ref			ref			ref			ref		
A steady partner	**2.15**	1.90 – 2.43	0.000	**2.08**	1.83 – 2.36	0.000	**1.68**	1.46 – 1.93	0.000	**1.67**	1.44 – 1.93	0.000
It’s complicated	**1.58**	1.30 – 1.92	0.000	**1.56**	1.28 – 1.91	0.000	**1.39**	1.11 – 1.74	0.005	**1.40**	1.11 – 1.76	0.005
**HIV diagnosis**												
No HIV diagnosis	ref			ref			ref			ref		
HIV diagnosed	**0.74**	0.67 – 0.82	0.000	**0.77**	0.69 – 0.86	0.000	**0.85**	0.73 – 0.98	0.027	0.87	0.74 – 1.01	0.074
**Place where last non-steady partner was met**												
Online	ref			ref			ref			ref		
Sex venue	**0.84**	0.71 – 0.99	0.038	0.85	0.72 – 1.02	0.076	**0.62**	0.50 – 0.76	0.000	**0.66**	0.53 – 0.82	0.000
Social venue	**1.22**	1.03 – 1.44	0.018	**1.22**	1.03 – 1.45	0.021	1.12	0.92 – 1.36	0.257	1.13	0.93 – 1.39	0.218
No sex with non-steady partners or not answered	1.17	0.99 – 1.38	0.074	**1.27**	1.06 – 1.51	0.008	1.07	0.86 – 1.33	0.532	1.23	0.98 – 1.53	0.074
**HIV status communication at last non-steady partner sex**												
No	ref			Ref			Ref			Ref		
Yes	**1.94**	1.71 – 2.21	0.000	**1.87**	1.63 – 2.14	0.000	**1.69**	1.48 – 1.94	0.000	**1.67**	1.45 – 1.92	0.000
**I find it easy to say ‘no’ to sex I don’t want**												
Agree or neutral	**1.35**	1.20 – 1.52	0.000	**1.27**	1.13 – 1.44	0.000	**1.26**	1.09 – 1.45	0.002	**1.17**	1.01 – 1.36	0.038
Disagree	ref			Ref			Ref			Ref		
**IH**												
Per score-point increase	**0.84**	0.81 – 0.88	0.000	**0.85**	0.82 – 0.89	0.000	**0.85**	0.81 – 0.89	0.000	**0.86**	0.82 – 0.90	0.000
**Cultural background (country-level)**												
Latin/Mediterranean	ref			ref			ref			ref		
Former socialist	**2.18**	1.48 – 3.23	0.000	**1.86**	1.23 – 2.81	0.008	**2.52**	1.59 – 3.99	0.000	**2.37**	1.49 – 3.76	0.000
Canada, North- and Central-West Europe	**3.26**	2.38 – 4.47	0.000	**2.78**	1.98 – 3.90	0.000	**3.80**	2.73 – 5.30	0.000	**3.04**	2.06 – 4.48	0.000
**Constant**	**2.06**	1.67 – 2.53	0.000	1.06	0.83 – 1.37	0.621	**2.43**	1.92 – 3.06	0.000	1.25	0.95 – 1.67	0.116
**Random component**												
country	0.40	0.23 – 0.69		0.11	0.05 – 0.25		0.55	0.33 – 0.92		0.16	0.08 – 0.32	

*CI* confidence interval, *OR* odds ratio, *aOR* adjusted odds ratio, ***Bold*** OR/aOR, statistically significant, *ref.* reference, *IH* short internalised homonegativity score (range 0–6)

### Multilevel multivariate analysis of factors associated with gonorrhoea PN

As for syphilis, lower values on the IH scale were also associated with higher PN rates after gonorrhoea diagnosis, as was higher perceived self-efficacy. Living in a steady partnership was positively associated with PN, albeit for gonorrhoea to a slightly smaller extent than for syphilis. Communication with the last non-steady partner(s) about HIV status was again positively associated with higher odds for PN. Contrasting with syphilis PN, the effect of HIV diagnosis on gonorrhoea PN was not significant. Men who had met their last non-steady partner in a gay sex venue were less likely to have notified their partners after having been diagnosed with gonorrhoea compared to men who had met their last non-steady partner(s) online.

Compared to syphilis, PN rates for gonorrhoea were not significantly affected by the French translation issue. Age 40 and older was associated with significant lower odds for partner notification.

The cultural background of the country where participants were living had an even larger impact on the willingness to notify partners as for syphilis (see Table 2).

## Discussion

We set out to examine PN rates after diagnosis of syphilis or gonorrhoea among MSM from 68 countries on four continents, and the impact of IH on PN. As we had hypothesized, IH was strongly associated with PN in all regions included in our analysis, for both syphilis and gonorrhoea. After grouping the 49 countries with large enough samples by historical and socio-cultural background in three areas, we were able to demonstrate a strong “dose–response” relationship between IH and PN in all three areas.

We had to exclude 19 countries from the analysis because the samples were too small. In most of the remaining 49 countries, a majority of respondents reported some kind of partner notification. The proportions of respondents reporting any kind of PN, i.e. who informed at least one partner, demonstrated large inter-country variability. More than half of those reporting any PN reported that they had informed all of their sex partners. However, the question on PN was not detailed enough to allow an exact calculation of the proportion of eligible partners who had been informed. If we compare reported PN rates from our study with a study reporting PN rates for MSM conducted in the Netherlands in 2010–2011 [35], the self-reported PN rates from EMIS-2017 participants living in the Netherlands were very similar to the PN rates in the Dutch study. This may suggest that self-reported PN-rates may refer primarily to notifiable partners and might neglect anonymous, non-notifiable partners.

Other factors associated with PN that had been identified by previous research such as partnership status, place where sexual partners met, and perceived sexual self-efficacy were confirmed by our analysis. We identified a history of HIV status communication with the last non-steady partner as an additional factor, probably as a surrogate for the ability to converse meaningfully about delicate topics with casual sex partners. Also, HIV diagnosis was negatively associated with PN. This negative association probably reflects an effect of HIV-associated stigma; persons diagnosed with HIV fear that PN for syphilis – more than for gonorrhoea – might take away their control over HIV status disclosure and fear of subsequent rejection based on HIV status [36, 37]. This fear may be based on the stronger association between syphilis and HIV diagnosis among MSM and the fact that both syphilis and HIV are usually diagnosed on a blood sample.

Another factor with a strong impact on PN that we identified by our analysis was the sociocultural background of the country where people lived regarding the concept of STI PN. It remains unclear what the basis for these sociocultural differences is. Empirically we identified three clusters of countries: cluster 1 was composed of Romanic speaking and other Eastern Mediterranean countries, Latin American countries, and the Philippines; cluster 2 was composed of the former socialist countries in eastern and central Europe; and cluster 3 was composed of North- and Central-West European countries and Canada. For cluster 1 with the lowest PN rates, we know from previous research that PN was not a routine part of STI case management until at least the late 1990s in the European Romanic speaking countries [28], and that there is low emphasis on PN in Latin America [29]. For cluster 2 we know that PN for STI was managed rigorously by a dedicated STI care system before the political transformation of these countries in the 1990s [26, 27]. STI care and control have changed considerably since then with greater emphasis on confidentiality, but still a dedicated STI care system is in place. An emphasis on PN may have partly survived at least in the public health sector, particularly since the region was struck by a major (heterosexual) syphilis epidemic in the 1990s as a consequence of the socioeconomic changes and a partial breakdown of public health systems during the transformation period [38–40]. Stronger social norms regarding syphilis PN in this region may also contribute to an equalization of the effect of IH on syphilis PN in the lower three quartiles as shown in Table 1. Cluster 3 comprises countries which placed traditionally a strong emphasis on PN and partly offer clinical PN support services like the Scandinavian countries, UK and Canada, but also include German-speaking and Benelux countries where this was hardly the case. This diversity of PN policies in cluster 3 may also partly explain the weaker dose–response effect of IH on syphilis PN.

One possibility could be that the differences between the clusters reflects different levels of PN-supportive counselling by sexual health care providers and gay community organizations in the three regions. It remains an area for further research to identify sociocultural factors and concepts that explain the differences in MSM PN rates across these three regions.

In terms of implications for the provision of sexual health care and counselling related to PN, our results suggest a sexual orientation affirmative approach, emphasizing gay community cohesion and mutual responsibility. Achieving a sense of community can reduce IH and foster PN at the same time. Stigma based on sexual orientation in healthcare is counterproductive and particularly has no place in sexual health care. This suggestion is matched by the conclusion of a systematic review on PN for STI in developing countries, which recommends counselling of index STI patients to raise awareness of PN and eliminate stigma and fear related to STIs [9].

### Strengths and limitations

A strength of our analysis is that we used the same method to collect data from 49 countries. Sexual orientation and PN are self-reported, avoiding biases due to differing capabilities of healthcare providers for eliciting this information in discussion with their clients. At the same time, relying on self-reported PN is also a limitation, because it can be biased by social desirability, recall, erroneous assessment which partners had been exposed to a transmission risk, and differences regarding STI diagnoses based on differences within and between countries in terms of STI screening practices. The surveys were convenience samples recruited on the internet and on apps. The samples may not be representative of all MSM in a country and/or region. PN rates are less reliable for smaller countries with limited numbers of respondents that could be asked about PN. Although we excluded countries with very small numbers of respondents, there was huge variation in the number of respondents by country. As a result, trends from countries with large numbers of respondents may be overrepresented in regional trends. We lack more detailed information on whether the issue of PN was addressed in the healthcare setting where the STI was diagnosed, which of the partners had subsequently been informed and if not, why not. The number of respondents that we could include in the multivariate analysis was halved in the EMIS dataset because EMIS participants were randomly assigned to answer the seven-question IH scale. Finally, PN outcomes and IH were collected in a cross-sectional study. We could neither assess causality, nor could we investigate the role of intermediary, more sociological concepts that would explain the causal pathway of IH further.

## Conclusions

In addition of confirming previously identified individual predictors of PN, we were able to demonstrate that IH is associated with PN among MSM across all countries included in our analysis. This finding contributes to the growing evidence of adverse health effects of homophobic policies and cultures. Reducing homophobia and ensuring inclusive policies in a welcoming environment may contribute to STI prevention and control.

## Supplementary Information

**Additional file 1: Supplemental Table S1.** Short Internalised Homonegativity Scale (SIHS).

**Additional file 2: Supplemental Table S2.** Self-reported rates of syphilis and gonorrhoea partner notification (PN) in EMIS and LAMIS countries, sorted by syphilis PN in three country groups.

## Data Availability

The EMIS-2017 and the LAMIS datasets used for this analysis have been obtained from the London School of Hygiene and Tropical Medicine (EMIS-2017) and from CEEISCAT, Barcelona (LAMIS) under data transfer agreements that prohibit to sharing the datasets publicly. Although we cannot make study data publicly accessible at the time of publication, all authors commit to make the data underlying the findings of the study available in compliance with the BMC Public Health Data Availability Policy. Data requests for the LAMIS dataset should be addressed to CEEISCAT/Fundació IGTP, Ctra. De Canyet s/n, 08916 Badalona – Catalonia, Spain: nlorente@coalitionplus.org, and the first author (MarcusU@rki.de). Data requests for the EMIS-2017 dataset should be addressed to the London School of Hygiene and Tropical Medicine Research Operations Office Data Management Lead: alex.hollander@lshtm.ac.uk, the first author (MarcusU@rki.de), and the Principal Investigator of EMIS-2017 (Peter.Weatherburn@lshtm.ac.uk). Individuals requesting data should present their research objective(s) and enclose a list of requested variables. To protect the confidentiality of participants, data sharing is contingent upon appropriate data handling and good scientific practice by the person requesting the data and should furthermore be in accordance with all applicable local requirements. The London School of Hygiene and Tropical Medicine administrative offices are located at Keppel Street, London WC1E 7HT, United Kingdom.

## Abbreviations

aOR: Adjusted odds ratio
CI: Confidence interval
EMIS: European MSM Internet Survey
HIV: Human immunodeficiency virus
IH: Internalised homonegativity
IP address: Internet protocol address
IQR: Interquartile range
LAMIS: Latin American MSM Internet Survey 2018
LR: Likelihood ratio
MSM: Men who have sex with men
PN: Partner notification
SD: Standard Deviation
SIHS: Short Internalised Homonegativity Scale
STI: Sexually transmitted infection
UK: United Kingdom

## References

[1] Low N, Broutet N, Adu-Sarkodie Y, Barton P, Hossain M, Hawkes S (2006). Global control of sexually transmitted infections. Lancet (London, England).

[2] European Centre for Disease Prevention and Control (2013). Public health benefits of partner notification for sexually transmitted infections and HIV.

[3] Clark JL, Perez-Brumer A, Salazar X. “Manejar la Situacion”: Partner Notification, Partner Management, and Conceptual Frameworks for HIV/STI Control Among MSM in Peru. AIDS Behav. 2015;19(12):2245–54. 10.1007/s10461-015-1049-3 PMC4586286 25821149

[4] Abraham T, Macauda M, Erickson P, Singer M. “And let me see them damn papers!” The role of STI/AIDS screening among urban African American and Puerto Rican youth in the transition to sex without a condom. AIDS Behav. 2010;15(7):1359–71. 10.1007/s10461-010-9811-z 20844945

[5] Gorbach PM, Galea JT, Amani B, Shin A, Celum C, Kerndt P, Golden MR. Don’t ask, don’t tell: patterns of HIV disclosure among HIV positive men who have sex with men with recent STI practising high risk behaviour in Los Angeles and Seattle. Sex Transm Infect. 2004;80(6):512–7. 10.1136/sti.2004.010918 PMC1744943 15572626

[6] Harrison A, Wilkinson D, Lurie M (1997). From partner notification to partner treatment. S Afr Med J.

[7] Klisch SA, Mamary E, Diaz Olavarrieta C, Garcia SG (2007). Patient-led partner notification for syphilis: Strategies used by women accessing antenatal care in urban Bolivia. Soc Sci Med (1982).

[8] Lichtenstein B, Schwebke JR (2005). Partner notification methods for African American men being treated for trichomoniasis: a consideration of main men, Second Hitters, and Third Players. Med Anthropol Q.

[9] Alam N, Chamot E, Vermund SH, Streatfield K, Kristensen S (2010). Partner notification for sexually transmitted infections in developing countries: a systematic review. BMC Public Health.

[10] Coleman C, Lohan M (2007). Sexually acquired infections: do lay experiences of partner notification challenge practice?. J Adv Nurs.

[11] Fortenberry JD, Brizendine EJ, Katz BP, Orr DP (2002). The role of self-efficacy and relationship quality in partner notification by adolescents with sexually transmitted infections. Arch Pediatr Adolesc Med.

[12] Mimiaga MJ, Reisner SL, Tetu AM, Bonafide KE, Cranston K, Bertrand T, Novak DS, Mayer KH. Partner notification after STD and HIV exposures and infections: knowledge, attitudes, and experiences of Massachusetts men who have sex with men. Public Health Rep (Washington, DC : 1974). 2009;124(1):111–9. 10.1177/003335490912400114 PMC2602936 19413033

[13] Schwartz RM, Malka ES, Augenbraun M, Rubin S, Hogben M, Liddon N, McCormack WM, Wilson TE (2006). Predictors of Partner Notification for C. trachomatis and N. gonorrhoeae: An Examination of Social Cognitive and Psychological Factors. J Urban Health.

[14] Frost DM, Meyer IH (2009). Internalized Homophobia and Relationship Quality among Lesbians, Gay Men, and Bisexuals. J Couns Psychol.

[15] Knoble NB, Linville D (2012). Outness and relationship satisfaction in same-gender couples. J Marital Fam Ther.

[16] Zlotnick C, Kohn R, Keitner G, Della Grotta SA (2000). The relationship between quality of interpersonal relationships and major depressive disorder: findings from the National Comorbidity Survey. J Affect Disord.

[17] Mayfield W (2001). The development of an Internalized Homonegativity Inventory for gay men. J Homosex.

[18] Fassinger RE, Miller BA (1997). Validation of an Inclusive Model of Sexual Minority Identity Formation on a Sample of Gay Men. J Homosex.

[19] Pachankis JE, Hatzenbuehler ML, Mirandola M, Weatherburn P, Berg RC, Marcus U, Schmidt AJ (2017). The Geography of Sexual Orientation: Structural Stigma and Sexual Attraction, Behavior, and Identity Among Men Who Have Sex with Men Across 38 European Countries. Arch Sex Behav.

[20] Pachankis JE, Hatzenbuehler ML, Hickson F, Weatherburn P, Berg RC, Marcus U, Schmidt AJ (2015). Hidden from health: structural stigma, sexual orientation concealment, and HIV across 38 countries in the European MSM Internet Survey. AIDS.

[21] Ramirez-Valles J (2002). The protective effects of community involvement for HIV risk behavior: a conceptual framework. Health Educ Res.

[22] Rosario M, Schrimshaw EW, Hunter J (2006). A model of sexual risk behaviors among young gay and bisexual men: longitudinal associations of mental health, substance abuse, sexual abuse, and the coming-out process. AIDS Educ Prev.

[23] Berg RC, Munthe-Kaas HM, Ross MW (2016). Internalized Homonegativity: A Systematic Mapping Review of Empirical Research. J Homosex.

[24] Davidson K, McLaren S, Jenkins M, Corboy D, Gibbs PM, Molloy M (2017). Internalized Homonegativity, Sense of Belonging, and Depressive Symptoms Among Australian Gay Men. J Homosex.

[25] Mimiaga MJ, Reisner SL, Tetu AM, Cranston K, Bertrand T, Novak DS, Mayer KH (2009). Psychosocial and behavioral predictors of partner notification after HIV and STI exposure and infection among MSM. AIDS Behav.

[26] Platt L, Mc Kee M (2000). Observations of the management of sexually transmitted diseases in the Russian Federation: a challenge of confidentiality. Int J STD AIDS.

[27] Renton AM, Borisenko KK, Tichonova LI, Akovbian VA (1999). The Control and Management of the Sexually Transmitted Diseases: A Comparison of the United Kingdom and the Russian Federation. Int J STD AIDS.

[28] Dehne KL, Riedner G, Neckermann C, Mykyev O, Ndowa FJ, Laukamm-Josten U (2002). A survey of STI policies and programmes in Europe: preliminary results. Sex Transm Infect.

[29] Garcia PJ, Benzaken AS, Galban E, members A-I (2011). STI management and control in Latin America: where do we stand and where do we go from here?. Sex Transm Infect.

[30] Weatherburn P, Hickson F, Reid DS, Marcus U, Schmidt AJ (2020). European Men-Who-Have-Sex-With-Men Internet Survey (EMIS-2017): Design and Methods. Sex Res Social Policy.

[31] Reyes-Díaz M, Celly A, Lorente N, et al. Latin American Internet Survey for Men who have Sex with Men (LAMIS-2018): design, methods and implementation. PloS One. 2022. 10.1371/journal.pone.0277518. 10.1371/journal.pone.0277518 PMC9671301 36395121

[32] Tran H, Ross MW, Diamond PM, Berg RC, Weatherburn P, Schmidt AJ (2018). Structural Validation and Multiple Group Assessment of the Short Internalized Homonegativity Scale in Homosexual and Bisexual Men in 38 European Countries: Results From the European MSM Internet Survey. J Sex Res.

[33] Lo SC, Reisen CA, Poppen PJ, Bianchi FT, Zea MC (2011). Cultural beliefs, partner characteristics, communication, and sexual risk among Latino MSM. AIDS Behav.

[34] Mimiaga MJ, Reisner SL, Bland SE, Driscoll MA, Cranston K, Isenberg D, VanDerwarker R, Mayer KH (2011). Sex parties among urban MSM: an emerging culture and HIV risk environment. AIDS Behav.

[35] van Aar F, Schreuder I, van Weert Y, Spijker R, Götz H (2012). Op de Coul E: Current practices of partner notification among MSM with HIV, gonorrhoea and syphilis in the Netherlands: an urgent need for improvement. BMC Infect Dis.

[36] Bilardi JE, Hulme-Chambers A, Chen MY, Fairley CK, Huffam SE, Tomnay JE (2019). The role of stigma in the acceptance and disclosure of HIV among recently diagnosed men who have sex with men in Australia: a qualitative study. PLoS One.

[37] Smith R, Rossetto K, Peterson BL. A meta-analysis of disclosure of one’s HIV-positive status, stigma and social support. AIDS Care. 2008;20(10):1266–75. 10.1080/09540120801926977 18608080

[38] Dehne KL, Pokrovskiy V, Kobyshcha Y, Schwartländer B (2000). Update on the epidemics of HIV and other sexually transmitted infections in the newly independent states of the former Soviet Union. AIDS.

[39] Gomberg MA, Akovbian VA. Resurgence of sexually transmitted diseases in Russia and eastern Europe. Dermatol Clin. 1998;16(4):659–62, ix−x. 10.1016/s0733-8635(05)70029-2 9891663

[40] Tichonova L, Borisenko K, Ward H, Meheus A, Gromyko A, Renton A (1997). Epidemics of syphilis in the Russian Federation: trends, origins, and priorities for control. Lancet (London, England).

