# Supplementary material for: Association of internalised homonegativity with partner notification after diagnosis of syphilis or gonorrhoea among men having sex with men in 49 countries across four continents
Source: BMC Public Health. 2023 Jan 3;23:8. doi: 10.1186/s12889-022-14891-2 (PMC9809117; doi:10.1186/s12889-022-14891-2)
Supplement: Supplementary file 1 — Additional file 1: Supplemental Table S1. Short Internalised Homonegativity Scale (SIHS). [file 12889_2022_14891_MOESM1_ESM.pdf]

Supplemental Table S1: Short Internalised Homonegativity Scale (SIHS)

There were seven statements respondents had to agree–disagree with that together form the Internalised Homonegativity Scale:

Q390. I feel comfortable in gay bars.
[7 point disagree-agree with does-not-apply]

Q391. Social situations with gay men make me feel uncomfortable.
[7 point disagree-agree with does-not-apply]

Q392. I feel comfortable being seen in public with an obviously gay person.
[7 point disagree-agree with does-not-apply]

Q393. I feel comfortable discussing homosexuality in a public situation.
[7 point disagree-agree with does-not-apply]

Q394. I feel comfortable being a homosexual man.
[7 point disagree-agree with does-not-apply]

Q395. Homosexuality is morally acceptable to me.
[7 point disagree-agree with does-not-apply]

Q396. Even if I could change my sexual orientation, I wouldn't.
[7 point disagree-agree with does-not-apply]

Taken together, the seven items Q390 –Q396 form a single scale measuring 'internalised homonegativity'. The score has been computed by: rescaling the items from 1 –7 to 0–6; reversing the scores for items Q390, Q392, Q393, Q394, Q395 and Q396; and taking the mean score for the seven items. The scale therefore ranges from 0 to 6. Scores can only be computed if all seven items were rated. Cases responding 'Does not apply to me' or with missing data for any of the seven items do not have an internalised homonegativity score.

See also: Tran H, Ross MW, Diamond PM, Berg RC, Weatherburn P, Schmidt AJ. Structural Validation and Multiple Group Assessment of the Short Internalized Homonegativity Scale in Homosexual and Bisexual Men in 38 European Countries: Results From the European MSM Internet Survey. *J Sex Res*. **2018**; 55(45):617–629. doi: 10.1080/00224499.2017.1380158.

https://www.emis-project.eu/questionnaires-2017/

Supplement to: Marcus U, Jonas K, Berg R, Veras MA, Caceres CF, Casabona J, Schink SB, Schmidt AJ. Association of internalised homonegativity with partner notification after diagnosis of syphilis or gonorrhoea among men having sex with men in 49 countries across four continents. *BMC Public Health*. **2022**
