# Supplementary material for: Association of internalised homonegativity with partner notification after diagnosis of syphilis or gonorrhoea among men having sex with men in 49 countries across four continents
Source: BMC Public Health. 2023 Jan 3;23:8. doi: 10.1186/s12889-022-14891-2 (PMC9809117; doi:10.1186/s12889-022-14891-2)
Supplement: Supplementary file 2 — Additional file 2: Supplemental Table S2. Self-reported rates of syphilis and gonorrhoea partner notification (PN) in EMIS and LAMIS countries, sorted by syphilis PN in three country groups. [file 12889_2022_14891_MOESM2_ESM.pdf]

Supplemental Table S2: Self-reported rates of syphilis and gonorrhoea partner notification (PN) in EMIS and LAMIS countries, sorted by syphilis PN in three country groups

		Syphilis partner notification						Gonorrhoea partner notification					
	Country	None	Yes, some	Yes, all	I don't remember	Total	Valid %	None	Yes, some	Yes, all	I don't remember	Total	Valid %
North- & Central-western European countries & Canada	Canada	18	58	124	7	207	91.0%	49	141	234	8	432	88.4%
	United Kingdom	38	143	168	10	359	89.1%	121	346	446	22	935	86.7%
	Netherlands	25	76	97	2	200	87.4%	36	166	168	5	375	90.3%
	Norway	6	12	25	1	44	86.0%	9	62	56	2	129	92.9%
	Switzerland[1]	14	45	35	2	96	85.1%	27	69	61	4	161	82.8%
	Austria	12	34	33	1	80	84.8%	13	38	52	3	106	87.4%
	Finland	3	6	9	0	18	83.3%	7	9	29	0	45	84.4%
	Belgium[1]	21	53	50	1	125	83.1%	23	50	47	4	124	80.8%
	Ireland	11	28	22	5	66	82.0%	38	70	71	6	185	78.8%
	Sweden	10	15	28	0	53	81.1%	9	56	83	0	148	93.9%
	Germany	147	282	308	16	753	80.1%	185	363	397	27	972	80.4%
	Denmark	10	24	16	2	52	80.0%	13	52	49	1	115	88.6%
Former socialist countries	Slovakia	1	3	7	1	12	90.9%	1	2	15	1	19	94.4%
	Hungary	8	16	33	1	58	86.0%	13	8	30	1	52	74.5%
	Czech Republic	8	15	26	1	50	83.7%	11	17	38	4	70	83.3%
	Poland	28	48	89	8	173	83.0%	27	27	43	6	103	72.2%
	Serbia	5	6	13	0	24	79.2%	1	4	5	0	10	90.0%
	Romania	11	11	25	5	52	76.6%	10	7	18	3	38	71.4%
	Slovenia	4	4	9	0	17	76.5%	4	5	8	0	17	76.5%
	Bulgaria	9	15	14	3	41	76.3%	9	9	11	1	30	69.0%
	Russia	63	59	77	9	208	68.3%	40	31	50	5	126	66.9%
	Croatia	4	4	3	0	11	63.6%	0	9	11	0	20	100.0%
	Ukraine	11	5	10	2	28	57.7%	3	7	10	2	22	85.0%
Latin-American/Mediterranean countries & the Philippines	Greece	26	41	30	2	99	73.2%	10	23	32	1	66	84.6%
	Turkey	14	20	15	2	51	71.4%	42	41	31	6	120	63.2%
	Italy*	114	131	151	8	404	71.2%	65	84	93	5	247	73.1%
	Spain	167	183	207	12	569	70.0%	191	203	218	24	636	68.8%
	Portugal	53	60	54	4	171	68.3%	33	42	66	8	149	76.6%
	Venezuela	28	25	35	1	89	68.2%	11	12	7	1	31	63.3%
	France[1]	13	11	16	3	43	67.5%	8	15	16	2	41	79.5%
	Panama	18	18	18	1	55	66.7%	15	8	7	1	31	50.0%
	Israel	12	9	14	0	35	65.7%	23	28	28	0	79	70.9%
	Brazil	653	549	657	57	1,916	64.9%	318	172	266	17	773	57.9%
	Argentina	138	112	111	12	373	61.8%	77	38	59	4	178	55.7%
	Costa Rica	26	21	19	2	68	60.6%	24	14	15	2	55	54.7%
	Guatemala	25	17	19	5	66	59.0%	16	12	18	2	48	65.2%
	Nicaragua	5	4	3	1	13	58.3%	9	3	0	0	12	25.0%
	Peru	51	43	24	6	124	56.8%	49	19	24	4	96	46.7%
	Chile	141	90	92	11	334	56.3%	88	39	50	6	183	50.3%
	Colombia	296	193	180	28	697	55.8%	177	97	98	10	382	52.4%
	Cyprus	4	1	4	1	10	55.6%	2	6	3	0	11	81.8%
	El Salvador	16	10	10	2	38	55.6%	6	0	6	0	12	50.0%
	Mexico	320	185	166	34	705	52.3%	229	130	124	13	496	52.6%
	Ecuador	35	14	24	2	75	52.1%	25	14	11	4	54	50.0%
	Honduras	11	7	4	1	23	50.0%	4	4	7	0	15	73.3%
	Uruguay	8	4	4	2	18	50.0%	4	7	6	0	17	76.5%
	Paraguay	38	14	18	4	74	45.7%	8	3	4	3	18	46.7%
	Philippines	30	14	6	2	52	40.0%	47	21	42	6	116	57.3%
	Bolivia	30	6	6	3	45	28.6%	19	14	7	2	42	52.5%
Countries excluded due to small numbers	Estonia[2]	1	0	3	0	4		1	0	2	0	3	
	Latvia[2]	2	1	3	1	7		0	0	3	0	3	
	Lithuania[2]	1	1	1	1	4		0	0	1	0	1	
	Luxembourg[2]	0	2	0	1	3		1	0	3	0	4	
	Malta[2]	0	5	3	1	9		3	8	6	0	17	
	Iceland[2]	0	3	0	0	3		0	3	3	0	6	
	Bosnia i H[2]	0	0	0	0	0		0	1	0	0	1	
	North Macedonia[2]	1	0	0	0	1		0	1	0	0	1	
	Albania-Kosovo-	0	1	2	0	3		2	3	2	0	7	
	Belarus[2]	5	0	3	0	8		3	6	4	2	15	
	Lebanon[2]	1	2	2	0	5		3	2	7	0	12	
	Moldova[2]	6	14	8	1	29		1	3	1	0	5	
	Suriname[2]	3	5	4	0	12		1	2	0	0	3	
	**Total**	**2,759**	**2,778**	**3,137**	**288**	**8,962**	**68.2%**	**2,164**	**2,626**	**3,202**	**228**	**8,220**	**72.9%**

[1]French questionnaire excluded; [2]Fewer than ten respondents with diagnosed syphilis or gonorrhoea

Supplement to: Marcus U, Jonas K, Berg R, Veras MA, Caceres CF, Casabona J, Schink SB, Schmidt AJ. Association of internalised homonegativity with partner notification after diagnosis of syphilis or gonorrhoea among men having sex with men in 49 countries across four continents. *BMC Public Health*. **2022**
